# Supplementary material for: Genome Size, Chromosome Number and Morphological Data Reveal Unexpected Infraspecific Variability in Festuca (Poaceae)
Source: Genes (Basel). 2021 Jun 11;12(6):906. doi: 10.3390/genes12060906 (PMC8230830; doi:10.3390/genes12060906)
Supplement: Supplementary file 1 [file genes-12-00906-s001.zip › genes-1238813-supplementary.pdf]

Supplementary Table S1. Summary of morphological variation in *Festuca yvesii* subsp. *summilusitana* and *F. yvesii* subsp. *lagascae* according to ploidy level. Mean, minimum and maximum values, standard deviation and coefficient of variation (%) were calculated for each character and ploidy level. Statistical differences and post-hoc test among cytotypes are provided.

Character	Subspecies	Ploidy level	Min-Max	Media	Sd	CV (%)	*p*-value	Post-hoc test
**Culm length (cm)**		10*x*	16.20-51.00	33.45	9.89	29.57		
	subsp. *summilusitana*	12*x*	15.00-51.33	31.50	10.94	34.73		
		14*x*	23.71-50.00	34.21	7.34	21.46		
		6*x*	10.00-22.00	16.08	3.74	23.26	< 0.001	a
	subsp. *lagascae*	8*x*	11.50-35.10	23.27	6.87	29.52		b
		10*x*	20.17-32.00	26.86	4.46	16.60		b
**Leaf length (cm)**		10*x*	4.00-18.00	9.56	3.69	38.60	0.027	a
	subsp. *summilusitana*	12*x*	2.57-16.35	7.13	3.28	46.00		ab
		14*x*	3.50-13.00	7.00	2.37	33.86		b
		6*x*	2.00-8.00	3.87	1.58	40.83	0.008	a
	subsp. *lagascae*	8*x*	1.97-9.43	4.87	1.97	40.45		ab
		10*x*	4.00-9.30	6.65	1.75	26.32		b
**Leaf diameter (mm)**		10*x*	0.66-1.10	0.82	0.12	14.63		
	subsp. *summilusitana*	12*x*	0.67-1.05	0.87	0.11	12.64		
		14*x*	0.70-1.13	0.86	0.10	11.63		
		6*x*	0.55-0.81	0.67	0.08	11.94	< 0.001	a
	subsp. *lagascae*	8*x*	0.60-1.15	0.85	0.14	16.47		b
		10*x*	0.65-0.93	0.83	0.11	13.25		b
**Auricle length (mm)**		10*x*	0.10-0.40	0.22	0.08	36.36		
	subsp. *summilusitana*	12*x*	0.10-0.50	0.22	0.09	40.91		
		14*x*	0.10-0.60	0.24	0.11	45.83		
		6*x*	0.05-0.20	0.13	0.05	38.46	< 0.001	a
	subsp. *lagascae*	8*x*	0.10-0.64	0.31	0.14	45.16		b
		10*x*	0.15-0.45	0.27	0.11	40.74		b
**Spikelet length (mm)**		10*x*	7.00-11.00	8.71	0.99	11.37		
	subsp. *summilusitana*	12*x*	7.20-12.00	9.22	1.07	11.61		
		14*x*	7.90-12.00	9.51	1.19	12.51		
		6*x*	5.50-8.75	7.24	0.83	11.46	< 0.001	a
	subsp. *lagascae*	8*x*	6.50-10.00	8.11	0.95	11.71		b
		10*x*	7.95-9.70	8.63	0.58	6.72		b
**Flowers number/spikelet**		10*x*	4-6	5.08	0.79	15.55	0.048	a
	subsp. *summilusitana*	12*x*	4-6	4.81	0.82	17.05		ab
		14*x*	4-6	4.48	0.61	13.62		b
		6*x*	3-5	4.39	0.76	17.31		
	subsp. *lagascae*	8*x*	3-6	4.81	0.73	15.18		
		10*x*	4-6	4.84	0.76	15.70		
**Upper glume length (mm)**		10*x*	2.60-5.63	4.39	0.59	13.44	0.006	a
	subsp. *summilusitana*	12*x*	4.10-6.40	4.83	0.47	9.73		b
		14*x*	4.00-6.80	4.89	0.61	12.47		b
		6*x*	3.50-4.70	4.03	0.42	10.42	0.003	a
	subsp. *lagascae*	8*x*	3.57-4.80	4.23	0.30	7.09		ab
		10*x*	4.25-5.00	4.55	0.25	5.49		b
**Lemma length (mm)**		10*x*	4.50-6.17	5.45	0.46	8.44	< 0.001	a
	subsp. *summilusitana*	12*x*	4.93-6.40	5.83	0.35	6.00		b
		14*x*	5.60-6.80	6.18	0.32	5.18		c
		6*x*	3.80-5.10	4.59	0.39	8.50	< 0.001	a
	subsp. *lagascae*	8*x*	4.60-5.97	5.21	0.38	7.29		b
		10*x*	4.95-5.78	5.31	0.27	5.08		b
**Awn lemma length (mm)**		10*x*	0.60-2.47	1.41	0.44	31.21		
	subsp. *summilusitana*	12*x*	1.10-2.50	1.71	0.36	21.05		
		14*x*	0.62-2.10	1.55	0.36	23.23		
		6*x*	1.00-2.50	1.73	0.43	24.86		
	subsp. *lagascae*	8*x*	1.07-2.80	1.86	0.44	23.66		
		10*x*	1.60-2.84	2.02	0.48	23.76		
**Anther length (mm)**		10*x*	2.20-3.20	2.66	0.28	10.53	< 0.001	a
	subsp. *summilusitana*	12*x*	2.30-3.40	2.88	0.30	10.42		b
		14*x*	2.50-3.80	3.07	0.28	9.12		b
		6*x*	1.60-3.00	2.52	0.36	14.29		
	subsp. *lagascae*	8*x*	2.07-3.10	2.56	0.27	10.55		
		10*x*	2.20-2.90	2.59	0.24	9.27		
**Inflorescence length (cm)**		10*x*	3.88-8.70	6.06	1.37	22.61		
	subsp. *summilusitana*	12*x*	3.30-8.80	6.14	1.49	24.27		
		14*x*	4.00-9.00	6.12	1.19	19.44		
	subsp. *lagascae*	6*x*	2.55-5.00	3.59	0.65	18.11	0.002	a
		8*x*	2.40-6.00	4.25	0.86	20.24		b

		10*x*	4.40-5.00	4.71	0.22	4.67		b
**First nude length (cm)**		10*x*	1.20-3.00	2.12	0.49	23.11		
	subsp. *summilusitana*	12*x*	0.90-4.20	2.09	0.68	32.54		
		14*x*	1.20-2.60	1.84	0.42	22.83		
		6*x*	0.50-1.30	0.96	0.18	18.75	< 0.001	a
	subsp. *lagascae*	8*x*	0.70-1.60	1.17	0.22	18.80		b
		10*x*	1.20-1.80	1.43	0.26	18.18		b
**Nodes number**		10*x*	7-14	10.24	2.19	21.39		
	subsp. *summilusitana*	12*x*	6-14	9.79	1.93	19.71		
		14*x*	8-13	9.81	1.17	11.93		
		6*x*	5-11	7.53	1.55	20.58		
	subsp. *lagascae*	8*x*	6-12	9.13	1.60	17.52		
		10*x*	8-10	9.00	0.63	7.00		
**Branches number**		10*x*	1-5	3.00	0.94	31.33		
	subsp. *summilusitana*	12*x*	2-5	2.79	1.08	38.71		
		14*x*	1-5	3.05	0.92	30.16		
		6*x*	1-3	2.18	0.64	29.36		
	subsp. *lagascae*	8*x*	1-5	2.84	1.19	41.90		
		10*x*	2-3	2.33	0.52	22.32		
**First branch length (cm)**		10*x*	1.50-3.70	2.91	0.34	11.68		
	subsp. *summilusitana*	12*x*	1.70-3.80	2.58	0.33	12.79		
		14*x*	1.80-3.50	2.59	0.37	14.29		
		6*x*	1.00-2.10	1.45	0.63	43.45	< 0.001	a
	subsp. *lagascae*	8*x*	1.30-2.80	1.97	0.62	31.47		b
		10*x*	1.50-2.40	2.08	0.50	24.04		b
**Seed length (mm)**		10*x*	-	-	-	-		
	subsp. *summilusitana*	12*x*	2.10-4.00	3.49	0.28	8.02	< 0.001	a
		14*x*	2.20-4.50	3.79	0.46	12.14		b
		6*x*	-	-	-	-		
	subsp. *lagascae*	8*x*	1.60-3.70	2.80	0.42	15.00	0.002	a
		10*x*	2.00-3.90	3.03	0.37	12.21		b
